# Digital Pathology Implementation in Private Practice: Specific Challenges and Opportunities

**DOI:** 10.3390/diagnostics12020529

**Published:** 2022-02-18

**Authors:** Diana Montezuma, Ana Monteiro, João Fraga, Liliana Ribeiro, Sofia Gonçalves, André Tavares, João Monteiro, Isabel Macedo-Pinto

**Affiliations:** 1 IMP Diagnostics, Edifício Trade Center do Bom Sucesso, 61, Sala 809, 4150-146 Porto, Portugal; ana.monteiro@impdiagnostics.com (A.M.); liliana.ribeiro@impdiagnostics.com (L.R.); sofia.goncalves@impdiagnostics.com (S.G.); andre.tavares@impdiagnostics.com (A.T.); joao.monteiro@impdiagnostics.com (J.M.); isabel.macedo.pinto@impdiagnostics.com (I.M.-P.); 2 Cancer Biology & Epigenetics Group, Research Center of IPO Porto (CI-IPOP)/RISE@CI-IPOP (Health Research Network), Portuguese Oncology Institute of Porto (IPO Porto)/Porto Comprehensive Cancer Center (Porto.CCC), R. Dr. António Bernardino de Almeida, 4200-072 Porto, Portugal; 3 Doctoral Programme in Medical Sciences, School of Medicine & Biomedical Sciences, University of Porto (ICBAS-UP), Rua Jorge Viterbo Ferreira 228, 4050-513 Porto, Portugal; 4 Department of Pathology, Portuguese Oncology Institute of Porto (IPO Porto), R. Dr. António Bernardino de Almeida, 4200-072 Porto, Portugal; joao.fraga89@hotmail.com

**Keywords:** digital pathology, WSI, LIS, artificial intelligence, routine diagnosis

## Abstract

Digital pathology (DP) is being deployed in many pathology laboratories, but most reported experiences refer to public health facilities. In this paper, we report our experience in DP transition at a high-volume private laboratory, addressing the main challenges in DP implementation in a private practice setting and how to overcome these issues. We started our implementation in 2020 and we are currently scanning 100% of our histology cases. Pre-existing sample tracking infrastructure facilitated this process. We are currently using two high-capacity scanners (Aperio GT450DX) to digitize all histology slides at 40×. Aperio eSlide Manager WebViewer viewing software is bidirectionally linked with the laboratory information system. Scanning error rate, during the test phase, was 2.1% (errors detected by the scanners) and 3.5% (manual quality control). Pre-scanning phase optimizations and vendor feedback and collaboration were crucial to improve WSI quality and are ongoing processes. Regarding pathologists’ validation, we followed the Royal College of Pathologists recommendations for DP implementation (adapted to our practice). Although private sector implementation of DP is not without its challenges, it will ultimately benefit from DP safety and quality-associated features. Furthermore, DP deployment lays the foundation for artificial intelligence tools integration, which will ultimately contribute to improving patient care.

## 1. Introduction

Digital pathology (DP) is gaining momentum worldwide as an innovative technology associated with improved laboratory efficiency and productivity. A progressive growth in DP deployment in many laboratories across the globe is taking place, but, in spite of this, real world data indicate that a fully digital transition has been accomplished in only a minority of pathology departments [1,2,3]. Moreover, most of the successful implementations reported in the literature concern public health laboratories and hospitals [1,4,5,6,7,8]. The reasons for low DP adoption in private practice laboratories are mostly related to the initial high costs of implementation, necessary workflow adjustments and pathologists’ receptivity. This is counterbalanced with future prospects of laboratory expenses reduction [8,9], easy remote access to cases and simple web-based case consultation by expert colleagues and improved data security [10,11]. In addition, already available digital tools to assist diagnosis (such as easy measuring, pinpointing or annotating relevant areas, etc.) can facilitate some pathologists’ tasks. Furthermore, the possibility to digitize glass slides enables the advent of artificial intelligence (AI) tools in pathology, which will be key in aiding pathologists in analysing and interpreting high-volume data [12]. The use of AI in pathology has clear potential, as recently demonstrated by the recent US Food and Drug Administration (FDA) approval of Paige Prostate, an AI-based pathology product for in vitro diagnostic use in detecting prostate cancer in biopsies [13]. Moreover, state-of-the-art AI approaches can be used for advanced tasks, including survival and therapy response prediction, which, if rigorously validated, can enhance clinical decision-making in the future [14]. Herein, we outline the roadmap of our implementation and address the specific hurdles of DP deployment in a private setting. We suggest how to overcome these issues so as to fully benefit from all the advantages and opportunities of DP.

## 2. Materials and Methods

### 2.1. Our Laboratory

IMP Diagnostics is composed of two laboratories, a central headquarters, based in Porto, and a Lisbon facility. It is a high-volume laboratory, having handled, in 2021, around 215,000 cases. These corresponded to 108,478 histology cases, 90,482 cytology samples and 18,085 molecular tests. The histology cases corresponded to a total of 296,814 slides (H&Es and immunostains). We receive cases not only from Portugal but also from other countries, namely Angola, Cape Verde Islands and Mozambique. There are currently 20 pathologists (4 full-time) and two dermatologists (with dermatopathology subspecialisation) working at our institution. We are a comprehensive private laboratory and not only provide pathology diagnostic services, but we also have a Research and Development (R&D) department, currently focusing on computational pathology projects.

### 2.2. Information Technology Infrastructure and Tracking System

Our Laboratory Information System (LIS) and sample tracking software are from GestPath (Esblada Medical, Barcelona, Spain). GestPath is a pathological anatomy process management system that digitizes all workflows, covering all areas and users of the service. Our lab already had an integrated 2D-barcode based tracking system, since 2016, which is mandatory for fully DP implementation. This enables adequate sample tracking in every step of the workflow. Patient requests and information arrive to the laboratory in two ways: paper request or direct digital link with the clinic/hospital. Regarding the paper requests, we scan these in order to make them available to consult from the LIS in the near future.

### 2.3. Imaging, Server and Storage Technology

We have two Aperio GT450DX Scanners by Leica Biosystems. These scanners each have a 450-slide capacity and enable brightfield applications and digitizing at 40× equivalent resolution (0.26 μm/pixel). The scanning in our laboratory includes standard H&E and special histochemical and immunohistochemical stains. We do not digitize immunofluorescence or cytology slides. The Image Managing System (IMS) we use is the Aperio eSlide Manager WebViewer viewing software by Leica Biosystems. Our DP server is a ProLiant DL380 Gen10, with 2× CPU Intel Xeon Silver 4208 CPU-2.10 GHz and 64 GB RAM, running Aperio eSlide Manager virtualized using 16 vCPU and 32 GB of RAM. Our data storage is from NetApp FAS2700 Series (FAS2750) and has 600 TB raw capacity plus 9.7 TB Flash Cache. We have 1 Gigabit per second (Gbps) internal and external client networks and the connection between the server and our internal network is 10 Gbps. Additionally, we have acquired a 2× CPU Intel DL Boost, 4× NVIDIA Tesla Volta V100, 384 GB RAM server for research purposes. For now, we are storing all the digitized slides (as well as the corresponding physical slides) and we do not apply any compression to the files. Regarding the glass slides, we follow the Best Practices Manual of Anatomical Pathology (defined in Portuguese law) and store them for at least 10 years (if malignant) or ≥5 years (other conditions). All tissue blocks are stored for 10 years at least. To date there are no rules in Portuguese law regarding the storage of digital pathology images, but we are expecting to keep in line with physical storage.

### 2.4. Pathologists’ Workstations

We currently have eight available workstations in the Porto laboratory. Most pathologists work part time, so all of them have access to the workstations during their work period. We mainly have two types of workstations (with some variations):

1. Workstation HP Z2 G5 Tower, Intel i7-10700, 16 GB RAM, 512 GB SSD; Radeon Pro W5500 graphic card; Monitor HP Z24N G3 24” (for reporting on LIS) and LG Clinical Monitor LED IPS 27” 16:9 8MP 4K 27HJ712C (for WSI viewing);
2. HP ProOne 600 AIO, Intel Core i5-9500, 8 GB RAM, 256 GB SSD; Intel UHD Graphics 630 graphic card; LCD wide screen FHD IPS 21,5” (for reporting on LIS) and LG Clinical Monitor LED IPS 27” 16:9 8MP 4K 27HJ712C (for WSI viewing).

### 2.5. LIS and IMS Integration

To allow for this integration, an initial step of requirements definition and vendor negotiation was undertaken. Bidirectional communication between LIS and IMS was implemented allowing the continuous exchange of information between the two systems. Communication between the LIS and the IMS is performed by means of a Health Level 7 messaging protocol. As pathologists are expected to mostly open the case images through the LIS, icons were created on the LIS screen to open the associated paper requests (if available), to call up the specific case images on the IMS. The worklist enables the pathologists to recognize completely digitized cases from only partially digitized ones (there is a specific icon which appears transparent in pending cases and that turns opaque when the case is completely digitized). In addition, an option for pathologists to request re-digitization or physical glass slides retrieval when needed is also being created.

### 2.6. Quality Control

As a way to test the DP system, before full implementation, we took advantage of our R&D department’s simultaneous AI project in colorectal cancer samples [15] and we evaluated the quality of 2963 digitized slides (1664 archive cases and 1299 routine cases). We divided the errors by those detected by the scanners and those detected in the subsequent pathology QC check. The quality control (QC) was performed by pathologists and biomedical scientists first by scanning the entire WSI at low magnification (4–10×) and then zooming in at 40× in multiple areas.

### 2.7. Validation

It is recommended that pathologists go through a training and validation process to ensure adequate transition to DP. This process is not unsubstantial as it requires pathologists to familiarize themselves with a different workflow, learn to handle new software and diagnose from an on-screen image. We followed an adapted version of the Royal College Guidelines, consisting of two phases [16]. As many of our pathologists see more than one diagnostic area, for phase 1 of the validation process, we opted for a mixed initial archive validation set (15 to 20 cases) with a representation of the most commonly described pitfalls in analogue to DP transition [17]. These case sets were elaborated in a personalized manner to each pathologist or small group of pathologists with 2 to 3 diagnostic areas. Then, the pathologists started to assess their routine workflow digitally, checking the corresponding glass slides before case sign-out (validation phase 2). This takes a variable amount of time, according to each pathologist, as it depends on self-assessment, and it is the pathologist’s decision when to start to analyse digitized cases only.

## 3. Results

### 3.1. Implementation Track, Challenges and Opportunities

**Figure 1:**
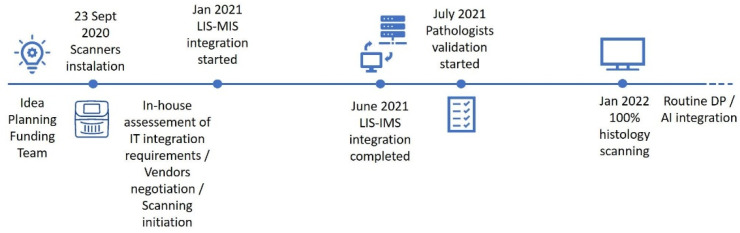
Digital implementation track timeline. DP, Digital Pathology; IMS, Image Managing System; LIS, Laboratory Information System.

Although we can pinpoint the kick-off of our implementation to the scanners installation (23 September 2020), the process began much earlier, around 2018, from the initial idea, to planning the project’s feasibility, raising funds and assembling a team (Figure 1). Scanner installation was followed by an in-house assessment of our requirements for IT integration and initial vendor negotiation, with the LIS–IMS integration kick-off starting in January 2021. The integration process then took around 5 to 6 months to be fully completed. Concomitantly, an initial test scanning phase was undertaken (digitizing 2963 slides, 44% archive material and 56% routine), followed by the incremental digitization of the routine workload. We have opted to begin by scanning single subspecialty areas and then scaling up to full digitization; around 110,000 histology slides have been digitized so far. The median scan time during the test phase was 98 s per slide, and the median size file was 1077 megabytes (MB). After the test phase, we optimized fragment placement within the slide (closer positioning of the fragments enabling narrower areas for digitization) and median scanning times and file size improved (74 s/slide and 792 MB median file dimension). In July 2021, we started pathologists’ validation, which is still ongoing, and by January 2022, we reached 100% histology digitization. Our implementation track is further discussed in the next section.

The main challenges encountered in DP implementation in our private practice were the high initial investment, difficulty to reorganize the lab workflow to include the scanning steps and little time availability of pathologists to engage in the initial learning phase. Moreover, although DP deployment in the private setting has its challenges, it also shows opportunities: easier and faster case delivery to the pathologists (which is extremely relevant for us, as we have two Porto buildings, as well as two laboratories in different cities, and physical slides need to be transported between sites); simpler case sharing between colleagues located in different places; and enabling working from home (for example, we have recently been able to hire a pathologist that is located in a different city, since he can easily work remotely). At this time, we still perform manual case distribution to subspecialized pathologists, but DP can also enable using computer-aided solutions, namely, to perform automatic case distribution. As we are a private laboratory with a Research and Development Unit, conducting studies in AI solutions for pathology, implementing DP was paramount. This may also be the case for other institutions/departments with an interest in investigation and development in this field. The main challenges and opportunities are outlined in Table 1 and are further addressed in the Discussion section.

### 3.2. Quality Control

**Figure 2:**
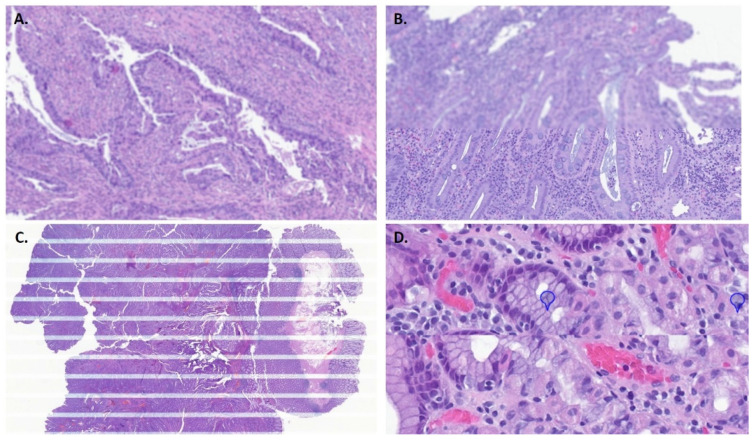
Errors detected in pathology quality control. (**A**). Out of focus. (**B**). Out of focus horizontal band. (**C**). Striping; (**D**). Stitch error/mismatch.

Common errors detected by the scanner included an inability to read the QR code on the slide and image quality issues and tilted slides (the scanner detects slides incorrectly inserted in the rack). Rarely, internal errors were signalized when the scanner was unable to pull out the slide from the rack or insert it back in, causing the scanning process to stop. During our test phase, 46 out of 2172 WSIs, in which this information was available, showed an error detected by the scanners (2.1%), with “skipped barcode” being the most common (25 cases). Regarding the other error types presented by the scanners: 14 “low image quality” cases (14/46 cases); 3 “tilted slide” (3/46 slides); 2 “no tissue” (2 cases, in which a minute fragment was not detected by the scanner); and 2 “internal errors”. In the ensuing manual pathology QC, the most commonly encountered issues were out of focus areas (which could vary between only focal areas to extensive ones), striping (horizontal stripes are seen across the image) and stitch error/mismatch (most WSI scanners capture contiguous images from the glass slide as patches and these sub-images are then put together to create the WSI. Sometimes this process can result in misalignment between the image patches or visible striping). In Figure 2, examples of errors detected during pathology QC are shown: 545 of 2963 WSIs had issues detected by the pathology QC (18.4%), but the majority of these corresponded to only small out-of-focus areas (440; 80.7%), with no probable impact in case diagnosis. As such, more relevant issues were seen in 105 slides (3.5% of all the analysed slides). Most of these (70 slides) were cases with apparent pre-scanning hitches, such as bubbles or folds. So, regarding slides in pristine conditions, only 35 presented significant errors detected on path QC. An additional detected issue corresponded to cases of duplicate or non-read slides that were only detected during pathology QC, with no warning message from the scanner (59 cases, 2%).

### 3.3. Validation

We started the validation process in July 2021, first with only one pathologist to allow for team and system verification and adjustments without disrupting routine workflow. We then scaled the validation process to small groups of pathologists at a time. We decided to start with the Porto headquarters laboratory, and only after will we start Lisbon’s pathologists’ validation process. Regarding phase 1 of the validation process, it has taken longer than predicted: around one to two months. For pathologists who have already initiated validation, most are in the final stage: routine observation of scanned slides and confrontation with corresponding glass slides before case sign-out. In our experience, the most commonly reported difficulties have been difficulty to discriminate H. pylori in gastric biopsy samples, nuclear detail assessment and mitosis counting. Granulomas and fungi detection were also considered potential pitfalls.

## 4. Discussion

### 4.1. Implementation Track, Challenges and Opportunities

In Figure 1, we describe our implementation path. One major issue in private settings is the ability to fund such a high-stake investment. In our case, as our implementation is part of a wider innovation project, part of our deployment (circa 70%) was financed by the European Regional Development Fund through an Operational Programme for Competitiveness and Internationalization. Applying for external funding may help other institutions in the process of DP deployment. Although DP is described as cost-efficient, leading to time savings in workflow and costs reduction [8,18,19], there should be no doubt that implementing DP represents a significant initial expense, as well as ongoing costs. Importantly, the possibility of future integration with AI solutions and operating in a scalable economy will introduce additional value [20,21]. In our case particularly, since we have laboratories in different locations, further relevant savings are expected due to the decreased transport of slides/blocks between cities. Regarding our implementation timeline, we were caught in the COVID pandemic and, although this emphasized the value of easy remote access to the lab, it has negatively impacted our deployment, and some phases took longer than anticipated. After the scanners installation, and before the official IT integration request was made, it was necessary to first define our system requirements in-house and to negotiate our options with the vendors, which took around 2 to 3 months. The integration process between the LIS and IMS then took approximately 5 to 6 months, being concluded in June 2021. Since the LIS provider did not have prior experience of integration with our IMS vendor, it had to be customized and built from scratch. Another issue in this process was the fact that the visualization software was not fully optimized for the 4 K monitors and, as such, we had to lower monitor resolution to improve performance. This precluded taking full advantage of the 40× high quality digitization and it is still being addressed with vendors (it is expected to be fixed in the new version of the software). As stated by Stathonikos N. et al., the process towards digital implementation is, at times, a “rocky road” and, as such, some issues during this undertaking are to be expected [6]. Importantly, turnaround time for case sign-out in private practice is highly constrained, so the most difficult step of the process was probably to initiate full capacity digitization (achieved in January 2022), since we had to adjust our workflow to limit the delay caused by adding the scanning step to the process. The way to improve this was to maintain a sustained flow, loading both scanners continuously, and also to optimize pre-scanning bottleneck steps. We opted for an incremental rollout for DP, firstly just scanning subspecialty areas and then scaling up to full digitization. This gave us more time to address possible constraints and difficulties. We also tried to diminish cases to be digitized overnight, since if a significant error occurred, there would not be a way to fix it timely. As such, we currently digitize almost all slides during the day, leaving only small batches, if needed, at night. Each laboratory must estimate its needs before deciding which equipment to acquire. Being a high-volume facility, we need to scan a large volume of slides daily (around 1000 slides on average), so our choice of having two high throughput scanners was crucial. After the initial test phase, we also optimized fragment placement within the slide (closer positioning enabling narrower areas for digitization) and the average scanning time and slide file size diminished. Regardless, these values are just a pointer, and will be different across different labs, as they vary according to the sample types: slides with more material (surgical specimens or dispersed biopsy fragments in the slide) will result, as expected, in longer scanning times and heavier file sizes.

### 4.2. Quality Control

Most articles addressing digital QC report a low scanning error rate, usually around 1–1.5%%, and, at most, less than 5% [1,7,9,22]. However, most studies only report the errors detected by the scanner and not by visual assessment of WSI image quality. Different labs report different ways to perform pathology QC: from checking all WSIs [5], to a percentage of cases, or even not performing a WSI QC, since it takes a significant amount of time for technicians to execute and can be considered unnecessary if the error rate is negligible [1]. In our experience, significant focus errors were encountered in 3.5% WSIs during the manual pathology QC in the test phase. On further review, we realized that many of these slides had some pre-scanning issues, such as bubbles, folds, excess mounting medium, etc. Thus, it is extremely important to optimize pre-analytical steps. First of all, to ensure adequate slide labelling; in our lab the slides are engraved with the QR codes, which leads to less scanning errors (most of the registered “skipped barcode” cases happened in archive slides, with stick on labels). Another important step is to ensure the slides have correctly aligned coverslips and are free of excessive mounting media. We use an automated equipment (HistoCore SPECTRA ST, Leica Biosystems) to obtain consistent staining and coverslipping. Regardless, sometimes we still have small bubbles appearing or excess mounting medium and addressing this issue has been a continuous process with the vendor. Some authors advocate the use of film coverslippers to minimize these problems [7,20]. Other important aspects to tackle are to make sure slides are clean by waiting until slides are fully dry before loading them into the scanner, and to ensure that slides are placed flat into the scanner rack. Regarding duplicate and non-read cases (which occurred in 2% of analysed cases), it was found to be a random event, in which the scanner did not recognize a rack and would process it twice (duplicating the slides) or, alternatively, would not scan it. This happened in two test batches and has occurred again, randomly, and, unfortunately, not so uncommonly, during the routine implementation phase, requiring multiple technical interventions by the vendor until the problem was finally solved. Lastly, we would recommend choosing the scanner location wisely. For a lean streamline, locate the scanners in a way that allows for a continuous workflow within the technical laboratory. Additionally, make sure scanners are placed on a flat surface, with as little vibration as possible. A missed issue when we started implementing DP in our laboratory was that one of the scanners was placed between two joint tables, causing some instability and probably contributing to scanning disturbances. After the initial test phase, we decided to register all scanner detected errors and to perform occasional pathology QC in a percentage of cases (about 5–10% of the daily workflow, randomly selected), as a way to address any issues and to give feedback to vendors if significant errors happened. Despite this, we continue to adjust our quality control necessities as implementation proceeds, as it requires a balance between necessity and time availability to perform it. Additionally, our LIS system will allow the pathologists to request the re-digitization of any slide they consider low quality. The analysis of these reported cases will give us a best estimation of the true impact of scanning errors on diagnosis. We expect to be in line with other studies that state that re-scanning requests are infrequent and do not impact global turnaround time [22], but we must emphasize that the initial deployment phase has shown more quality issues than anticipated.

### 4.3. Validation

As previously stated, phase 1 of the validation process has taken pathologists longer than predicted: around one to two months. The fact that many pathologists work in the laboratory only part time, alongside a significant workload, made it difficult to evaluate the archive slide boxes and it was carried out over a gradual period of time. In fact, one of the biggest issues in deploying DP in a private laboratory is that many pathologists work only part time and the turnaround time for case sign-out is highly constrained. So, during the adjustment period, when a two-track observation (analogue and digital) is necessary, the pathologist’s efficiency can be negatively impacted (having to see the same case twice). This is a practical handicap of DP implementation in a private setting. One way to ease this during phase 2 of validation was that pathologists only had to assess a percentage of daily workload in both digital and glass slides, at their own pace. Additionally, realizing the added value DP can represent once it is fully implemented (such as possibility to work remotely, simplicity in second opinion requests, available digital tools to assist diagnosis and, in the near future, AI driven solutions) has facilitated the pathologists’ engagement to this initial validation phase. Furthermore, many pathologists report an easy and relatively fast learning curve, and, as such, the two-track period can be shortened and cause less disruption [23]. Even so, we have decided to maintain the parallel analogue and digital workflows for a period of about one year, in accordance with other implementation reports [4,5,6], to allow a smooth transition to routine use of DP. Moreover, as advised in the Royal College Guidelines [16,17], it will be up to each pathologist to decide when to abandon glass slides in favour of WSI visualization. Of note is that the two-track workflow, although allowing the pathologist to gain more confidence in DP, precludes all users from taking full advantage of DP benefits, since there is no immediate reduction in time spent and workload related to assembling and delivering glass slides to the pathologists [24]. Other laboratories must be aware of this when deciding their validation procedure. As previously stated, the most common reported difficulties have been difficulty to discriminate H. pylori in gastric biopsies (even when using immunostains, since the focus may be suboptimal in the surface area where most of the bacilli are observed); assessing nuclear detail and counting mitosis. Granulomas and fungi were also more difficult to see on digital versus conventional slides. These findings are in line with other literature reports [5,6,17]. We expect further practice and plan additional improvements to the visualization software that will diminish these issues, as it is currently necessary to check the corresponding physical slides in doubtful cases. Furthermore, future coupled computer-aided solutions have the potential to be noninferior or even superior to conventional microscopy regarding some of these issues: automatic counting mitosis or assessment of the presence of microorganisms, for example, could potentially be performed by robust AI algorithms, solving these current challenges.

## 5. Conclusions

We are currently digitizing 100% of our histology slides and pathologists are performing the validation process for routine diagnosis using WSIs. DP implementation in a private setting is not without its challenges and has specific difficulties that are important to draw attention to, namely the high costs of its deployment and the pathologists’ low time availability to engage in the initial learning phase. Scanning device selection should be based on planned use and budget and care should also be given to LIS–WSI integration and archive requirements. Despite this, we believe the benefits of DP in the long run will far exceed the initial handicaps in its deployment. DP provides lower costs associated with slides assembly, retrieval and transport; facilitates remote work and case consultation (enabling, for example, to hire pathologists from distant locations); and the use of digital tools to ease many diagnostic tasks. DP’s potential for improvement in patient safety, work quality and efficiency is, in itself, a sufficient argument for its widespread implementation [25]. The European Society of Digital and Integrative Pathology (ESDIP) has recently provided consensus-based recommendations for the implementation of a DP workflow for the Pathology Laboratory in a practical document that can further assist other practices to successfully deploy DP in Europe [26]. Additionally, future implementation of AI-based solutions will provide many advantages over traditional pathology, namely generating highly precise and consistent readouts that can assist pathologists in their daily decisions. After all, is there any pathologist who will not be happy to automate PD-L1 counting? We hope that DP implementation is seen in a holistic approach, as described by Betmouni S. [2], considering not only technology and pathology laboratories, but also the broad healthcare team and patients as potential beneficiaries.

## Figures and Tables

**Table 1:** Challenges and opportunities in DP deployment in private practice.

**Challenges**
▪ High investment in initial deployment and development. ▪ Necessity for workflow adjustments in the technical laboratory. ▪ Time constraints in case turnaround time in the private setting make initial learning phase more difficult for pathologists. ▪ Software and hardware glitches and malfunction are more prone to happen, comparing with conventional microscopy.
**Opportunities**
▪ Easy and fast delivery of cases to pathologists. ▪ Diminishes the need for physical slide transport (namely across different laboratories). ▪ Facilitates case sharing between colleagues in different locations. ▪ Enables easy case consultation by experts in other locations. ▪ Allows working from home and a more flexible schedule. ▪ Possibility to hire pathologists at different locations of the laboratory. ▪ Essential for AI and DP Research and Development projects. ▪ Will enable the use of Computer Aided Solutions in routine work.

## Data Availability

Not applicable.

## References

[1] Fraggetta F., Caputo A., Guglielmino R., Pellegrino M.G., Runza G., L’Imperio V.A. (2021). Survival Guide for the Rapid Transition to a Fully Digital Workflow: The “Caltagirone Example”. Diagnostics.

[2] Betmouni S. (2021). Diagnostic digital pathology implementation: Learning from the digital health experience. Digit. Health.

[3] Schüffler P.J., Geneslaw L., Yarlagadda D.V.K., Hanna M.G., Samboy J., Stamelos E., Vanderbilt C., Philip J., Jean M.H., Corsale L. (2021). Integrated digital pathology at scale: A solution for clinical diagnostics and cancer research at a large academic medical center. J. Am. Med. Inform. Assoc..

[4] Fraggetta F., Garozzo S., Zannoni G.F., Pantanowitz L., Rossi E.D. (2017). Routine Digital Pathology Workflow: The Catania Experience. J. Patho.l Inform..

[5] Stathonikos N., Nguyen T.Q., Spoto C.P., Verdaasdonk MA M., van Diest P.J. (2019). Being fully digital: Perspective of a Dutch academic pathology laboratory. Histopathology.

[6] Stathonikos N., Nguyen T.Q., van Diest P.J. (2021). Rocky road to digital diagnostics: Implementation issues and exhilarating experiences. J. Clin. Pathol..

[7] Retamero J.A., Aneiros-Fernandez J., Del Moral R.G. (2020). Complete Digital Pathology for Routine Histopathology Diagnosis in a Multicenter Hospital Network. Arch. Pathol. Lab. Med..

[8] Hanna M.G., Reuter V.E., Samboy J., England C., Corsale L., Fine S.W., Agaram N.P., Stamelos E., Yagi Y., Hameed M. (2019). Implementation of Digital Pathology Offers Clinical and Operational Increase in Efficiency and Cost Savings. Arch. Pathol. Lab. Med..

[9] Quigley J.C., Lujan G., Hartman D., Parwani A., Roehmholdt B., Van Meter B., Ardon O., Hanna M.G., Kelly D., Sowards C. (2021). Dissecting the Business Case for Adoption and Implementation of Digital Pathology: A White Paper from the Digital Pathology Association. J. Pathol. Inform..

[10] Jahn S.W., Plass M., Moinfar F. (2020). Digital Pathology: Advantages, Limitations and Emerging Perspectives. J. Clin. Med..

[11] Pallua J.D., Brunner A., Zelger B., Schirmer M., Haybaeck J. (2020). The future of pathology is digital. Pathol. Res. Pract..

[12] Bera K., Schalper K.A., Rimm D.L., Velcheti V., Madabhushi A. (2019). Artificial intelligence in digital pathology—New tools for diagnosis and precision oncology. Nat. Rev. Clin. Oncol..

[13] Food and Drug Administration. https://www.accessdata.fda.gov/cdrh_docs/pdf20/DEN200080.pdf..

[14] Echle A., Rindtorff N.T., Brinker T.J., Luedde T., Pearson A.T., Kather J.N. (2021). Deep learning in cancer pathology: A new generation of clinical biomarkers. Br. J. Cancer.

[15] Oliveira S.P., Neto P.C., Fraga J., Montezuma D., Monteiro A., Monteiro J., Ribeiro L., Gonçalves S., Pinto I.M., Cardoso J.S. (2021). CAD systems for colorectal cancer from WSI are still not ready for clinical acceptance. Sci. Rep..

[16] Royal College of Pathologists (2018). Best Practice Recommendations for Digital Pathology. https://www.rcpath.org/resourceLibrary/best-practicerecommendations-for-implementing-digital-pathology-pdf.

[17] Williams B.J., Treanor D. (2020). Practical guide to training and validation for primary diagnosis with digital pathology. J. Clin. Pathol..

[18] Baidoshvili A., Bucur A., van Leeuwen J., van der Laak J., Kluin P., van Diest P.J. (2018). Evaluating the benefits of digital pathology implementation: Time savings in laboratory logistics. Histopathology.

[19] Ho J., Ahlers S.M., Stratman C., Aridor O., Pantanowitz L., Fine J.L., Kuzmishin J.A., Montalto M.C., Parwani A.V. (2014). Can digital pathology result in cost savings? A financial projection for digital pathology implementation at a large integrated health care organization. J. Pathol. Inform..

[20] Eloy C., Vale J., Curado M., Polónia A., Campelos S., Caramelo A., Sousa R., Sobrinho-Simões M. (2021). Digital Pathology Workflow Implementation at IPATIMUP. Diagnostics.

[21] Hanna M.G., Ardon O., Reuter V.E., Sirintrapun S.J., England C., Klimstra D.S., Hameed M.R. (2021). Integrating digital pathology into clinical practice. Mod Pathol. Mod. Pathol..

[22] Hanna M.G., Reuter V.E., Ardon O., Kim D., Sirintrapun S.J., Schüffler P.J., Busam K.J., Sauter J.L., Brogi E., Tan L.K. (2020). Validation of a digital pathology system including remote review during the COVID-19 pandemic. Mod. Pathol..

[23] Retamero J.A., Aneiros-Fernandez J., Del Moral R.G. (2020). Microscope? No, Thanks: User Experience With Complete Digital Pathology for Routine Diagnosis. Arch. Pathol. Lab. Med..

[24] Evans A.J., Salama M.E., Henricks W.H., Pantanowitz L. (2017). Implementation of Whole Slide Imaging for Clinical Purposes: Issues to Consider From the Perspective of Early Adopters. Arch. Pathol. Lab. Med..

[25] Griffin J., Treanor D. (2017). Digital pathology in clinical use: Where are we now and what is holding us back?. Histopathology.

[26] Fraggetta F., L’Imperio V., Ameisen D., Carvalho R., Leh S., Kiehl T.R., Serbanescu M., Racoceanu D., Della Mea V., Polonia A. (2021). Best Practice Recommendations for the Implementation of a Digital Pathology Workflow in the Anatomic Pathology Laboratory by the European Society of Digital and Integrative Pathology (ESDIP). Diagnostics.

